# Perspective on the Integration of Diagnostic Algorithms for Fever Management

**DOI:** 10.1093/cid/ciad325

**Published:** 2023-07-25

**Authors:** Juvenal Nkeramahame, Piero Olliaro, Philip Horgan, Sabine Dittrich

**Affiliations:** Medical Affairs, FIND, Geneva, Switzerland; Medical Affairs, FIND, Geneva, Switzerland; International Severe Acute Respiratory and Emerging Infection Consortium, Pandemic Sciences Institute, University of Oxford, Oxford, United Kingdom; Medical Affairs, FIND, Geneva, Switzerland; Nuffield Department of Medicine, Big Data Institute, University of Oxford, Oxford, United Kingdom; Department of Medicine, Evidence & Impact - Oxford, Oxford, United Kingdom; Medical Affairs, FIND, Geneva, Switzerland; Center for Tropical Medicine and Global Health, Nuffield Department of Medicine, University of Oxford, Oxford, United Kingdom; Deggendorf Institute of Technology, European Campus Rottal Inn, Pfarrkirchen, Germany

**Keywords:** Antibiotic resistance, acute fever, diagnostics, diagnostic algorithms

## Abstract

The AMR Diagnostics Use Accelerator Program was established to address antimicrobial resistance. Here, we bring into broad perspective the findings and missed opportunities of the first phase of the program and look toward the second phase.

Acute febrile illnesses (AFIs) are a major public health problem and the reason for most outpatient clinic consultations at primary healthcare centers [1]. The causes of AFIs are usually diverse. Distinguishing between bacterial and viral infectious causes of nonmalarial fever using noninvasive sampling is inherently difficult due to the limited performance of diagnostics, presence of normal flora, and carriage, among other reasons. The challenge is present even in high-income countries but is more magnified in low- and middle-income countries where access to accurate diagnostics is limited. Even with the rollout of the World Health Organization’s (WHO’s) “test, treat, and track” policy on malaria, cautionary just-in-case antibiotic prescriptions are still common, more so among patients with AFIs who test negative for malaria, children aged <5 years, and patients who have respiratory tract infections [2–6].

This diagnostic uncertainty is a driver of antibiotic prescriptions for AFIs. Unnecessary antibiotic prescriptions contribute to a rise in antimicrobial resistance (AMR), which is a “silent pandemic” that in 2019 alone was associated with 4.95 million deaths globally and caused 1.27 million deaths globally [7]. Strategies to rationalize antibiotic use, reduce unnecessary antibiotic prescriptions, and address self-medication are needed to stem the growing problem. The ability to establish a correct diagnosis between viral and bacterial causes of fever is central to this response.

## FINDINGS FROM THE AMR DIAGNOSTICS USE ACCELERATOR STUDIES

From September 2020 to September 2021, FIND coordinated the conduct of three randomized controlled clinical trials in three African countries (Burkina Faso, Ghana, and Uganda) under the Advancing Access to Diagnostic Innovation Essential for Universal Health Coverage and AMR Prevention Program. The purpose was to evaluate the impact of commercially available diagnostic tests on antibiotic prescriptions among children, adolescents, and adults with AFIs at primary healthcare centers [8]. The point-of-care tests (POCTs) were selected based on local fever epidemiology and availability and were implemented in the context of diagnostic algorithms to guide antibiotic prescriptions. Training and communication packages were given to healthcare workers and patients/caregivers as part of the intervention in support of prescription adherence. There was heterogeneity across the countries and sites in terms of participant characteristics, adherence to the recommendations of the diagnostic algorithms, and the overall antibiotic prescriptions.

The algorithms and POCTs resulted in a 9% reduction in antibiotic prescriptions relative to standard practices. While the reduction may not appear substantial, this translates into 1 fewer antibiotic prescription for every 20 patients. Importantly, statistically significant reductions were observed in the most at-risk groups: children aged <5 years, patients with nonmalarial fevers, and those with respiratory symptoms. Patients who tested negative on a malaria rapid diagnostic test (nonmalarial fever) had the greatest reduction in antibiotic prescriptions of 30%. This effect was consistent across all 3 countries and in a fixed-effects meta-analysis (see Kapisi et al [9], Kiemde et al [10], Adjei et al [11], and Olliaro et al [12] in this supplement). The POCTs and diagnostic algorithms also reduced antibiotic prescriptions by 16% in children aged <5 years, by 23% in patients who had respiratory symptoms at presentation, and by 31% in those with respiratory symptoms and a malaria-negative test. These results reiterate the need for improved access to diagnostics and guidance for healthcare professionals for fever among these patient groups.

However, we are still seeing a high rate of antibiotic prescriptions among those with positive tests for malaria that could not be explained by the rates of bacterial coinfections (see Kapisi et al [9] in this supplement). In this case, markers such as C-reactive protein (CRP) cannot help since CRP levels are usually increased in the presence of malaria. Holistic approaches are needed as other factors such as the level of training of healthcare workers, patient and healthcare worker diagnostic uncertainty, health facility level in the health system, and adherence to recommendations contribute to this problem [4, 5, 13].

## ADHERENCE TO PRESCRIPTIONS

Adherence to prescriptions is an important part of the treatment pathway. Nested qualitative research in this study reveals that the reasons why patients do not adhere to prescriptions are many and varied but center around the ability of healthcare workers to communicate effectively with the patients so they understand the basic prescription instructions. Beyond this, persuasive communication can help address the various personal, social, and cultural factors that pull patients away from adherence but for which other reinforcing interventions are also needed. There are a number of other, more structural, issues that need a different type of intervention. These behavioral drivers were broadly consistent across the 3 countries and research sites, with some nuances (described in the other articles in this supplement). While qualitative and quantitative studies show that commonalities across different study sites exist, our work clearly shows the value of country-specific research and communication messages, which reinforces the fact that not one solution fits all. However, where qualitative assessment is not feasible and the settings are similar to our study sites, our findings can be used to develop a communication package that is based on the common themes, adapted for local language and culture.

Adherence to diagnostic test results and diagnostic algorithms by healthcare workers is equally important to reduce just-in-case antibiotic prescriptions. Adherence varies among healthcare workers, clinics, and countries, and this variability partly explains the differential impact of interventions [14]. Electronic algorithms or clinical decision support tools have the potential to reinforce adherence by healthcare workers and reduce antibiotic prescriptions for fever without negatively affecting clinical outcomes [15–18]. However, the challenges of implementing electronic algorithms, in addition to traditional/manual algorithms, such as limited computer skills, alignment with other clinic programs, internet access, healthcare worker perceptions, and workloads, must be taken into consideration [19]. Additionally, in many countries, there are existing algorithms for management of fever, for example, the WHO Integrated Management of Childhood Illnesses (IMCI) guidelines. The implementation of new algorithms must factor in existing guidance. For these studies, we integrated the IMCI guidance into diagnostic algorithms.

## FEVER MANAGEMENT IN THE AMR DX USE ACCELERATOR IN THE CONTEXT OF CORONAVIRUS DISEASE 2019

The coronavirus disease 2019 (COVID-19) pandemic disrupted many public health achievements including in the management of AFIs. In many countries, antibiotics such as azithromycin were used for the treatment of COVID-19 despite evidence against it [20–22]. In addition, diagnostics and treatment supply limitations drove more antibiotic prescriptions.

In 2021 at the height of the pandemic, we amended the diagnostic algorithms in the AMR Diagnostic Use Accelerator study to incorporate commercially available severe acute respiratory syndrome coronavirus 2 antigen-detecting POCTs. In an attempt to increase adherence to the diagnostic algorithm, in this second phase of the AMR Dx Use accelerator, we deployed electronic diagnostic algorithms to improve adherence to the recommendations of the diagnostic algorithm. The second phase of the project enrolled more than 11,000 participants (including those suspected to have mild COVID-19) in 4 countries (Burkina Faso, India, Nepal, and Uganda) and is at the analysis stage.

## CONSIDERATIONS FOR POLICY AND IMPLEMENTATION

While POCTs are integral to reducing and rationalizing antibiotic use, it is often difficult to determine what set of tests has the greatest yield in terms of reductions in unnecessary antibiotic prescriptions and cost, both from the clinical and public health perspectives. In the AMR Diagnostic Use Accelerator study, tests were performed at the discretion of healthcare workers and varied from country to country, making it complex to “tease out” the most appropriate “minimal set of tests” to implement in a given country, setting, or population.

Future research is needed to evaluate the impact of minimal packages of POCTs and how electronic tools can be used to improve adherence to diagnostic algorithms by healthcare workers. Most importantly, the differentiation of fever among children, patients with nonmalarial fever, and those with respiratory syndromes needs to take center stage in the management of AFIs.

Last, the implementation of diagnostic algorithms, whether paper-based or electronic, is complex. Other factors such as skills and the workload of healthcare workers must be taken into account. Holistic measures must be taken to ensure that human resources, infrastructure, and policies are in tandem with sustainability.

## Notes

***Author contributions.*** J. N. wrote the first draft. P. O., P. H., and S. D. reviewed and edited the manuscript. All authors were involved in revising the manuscript and approved the final version for submission.

***Financial support*.** The Phase I of the AMR Diagnostics Use Accelerator studies was funded by the Swiss Agency for Development and Cooperation, the Federal Ministry of Economic Cooperation and Development (Bundesministerium für Wirtschaftliche Zusammenarbeit und Entwicklung) [BMZ], and the Department for International Development, United Kingdom, now the Foreign, Commonwealth and Development Office, United Kingdom.

***Supplement sponsorship.*** This article appears as part of the supplement “Using Diagnostic Tools to Support Antimicrobial Stewardship and Improve Outcome in Resource-Limited Contexts,” sponsored by FIND.
